# A Study on the Characteristics of 304 Stainless Steel According to the Water Temperature Changes in Underwater Laser Beam Machining

**DOI:** 10.3390/ma16237463

**Published:** 2023-11-30

**Authors:** Jun Yeon Lee, Dong-Hyeon Kim, Young Tae Cho, Choon Man Lee

**Affiliations:** 1 Department of Smart Manufacturing Engineering, Changwon National University, 20, Changwondaehak-ro, Uichang-gu, Changwon-si 51140, Republic of Korea; 20227175@gs.cwnu.ac.kr (J.Y.L.); ytcho@changwon.ac.kr (Y.T.C.); 2 Mechatronics Research Center, Changwon National University, Changwon 51140, Republic of Korea

**Keywords:** underwater laser beam machining, heating system, heat-affected zone, mechanical property, 304 stainless steel

## Abstract

In underwater laser beam machining (ULBM), water provides a cooling effect by reducing the influence of the laser heat source, which makes ULBM more suitable for marking, cutting, and postprocessing than laser beam machining (LBM). Because the laser heat source not only affects the substrate temperature, but also heats the water, this study analyzes how the cooling effect occurs when water is heated. In this study, the heat-transformed zones in ULBM and heated underwater laser beam machining (HULBM) were improved by approximately 33% and 24%, respectively, compared to LBM at 400 W. In addition, the heat-affected zones in ULBM and HULBM improved by approximately 15% and 9%, respectively, compared to LBM. The hardness of ULBM and HULBM was higher than that of LBM. Based on these results, it was confirmed that water can reduce the effect of the laser heat source and improve the mechanical properties. Experiments will be conducted on the underwater laser beam machining of various substrates, such as Inconel718 and Ti-6Al-4V, in a future study. In addition, experiments will be conducted on the underwater laser beam machining of various substrates using a cooling system that can lower the temperature of water.

## 1. Introduction

The automobile and aerospace industries are constantly advancing, and research on postprocesses such as laser heat sources, plasma, and end mills has, therefore, been continuously conducted. After producing the parts, a postprocessing step is essential to improve the quality of the surface of the parts. Plasma and end-mill processes have disadvantages, such as a high initial cost, a low process efficiency, and tool damage. In contrast, the laser heat source can be adjusted to the appropriate power for various materials. Furthermore, if there is no failure of parts or equipment, they can be maintained semi-permanently without needing repair [1,2,3,4].

Laser beam machining (LBM) involves focusing a laser beam on a small area of a substrate surface and manufacturing parts. Owing to their high power density and energy efficiency, LBM processes have been utilized in various technologies such as marking, drilling, cutting, and microprocessing. However, during the LBM process in air, such as in the heat-affected zone (HAZ), cracks are often observed owing to high thermal gradients. In addition, the laser process efficiency is very low during LBM in air. To overcome these disadvantages, research has been conducted on liquid-assisted LBM, such as underwater laser beam machining (ULBM) [5,6].

**Figure 1:**
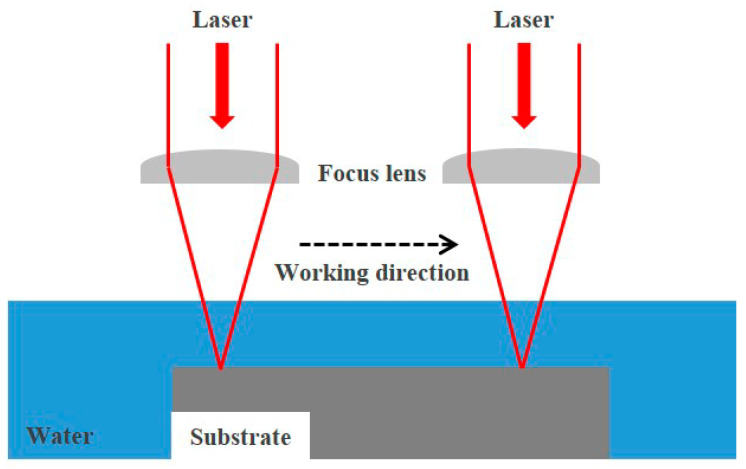
Conceptual diagram of underwater laser beam machining process.

ULBM utilizes water from the surface of the substrate as a coolant for LBM. Figure 1 illustrates a schematic of the ULBM process. ULBM has advantages in that the deformation of the substrate by the laser heat source is reduced, and the mechanical properties are improved by reducing the HAZ. However, the laser power is reduced by the absorption and transmission of the laser beam by water, and the bubbles generated during the process disturb the scan of the laser heat source. To solve these problems, research on underwater laser beam machining is important [7,8].

ULBM processes have been studied using different types of lasers and various materials. Maharjan et al. performed underwater laser ablation, utilizing a fiber laser, on bearing steel with respect to the surface morphology and microstructure [9]. Marla et al. investigated the fundamental mechanisms of underwater laser ablation using a Nd:YAG laser [10]. Tangwarodomnukun et al. reported underwater laser ablation with flowing water using a nanosecond pulse laser on a titanium alloy for cut quality and thermal damage [11].

**Figure 2:**
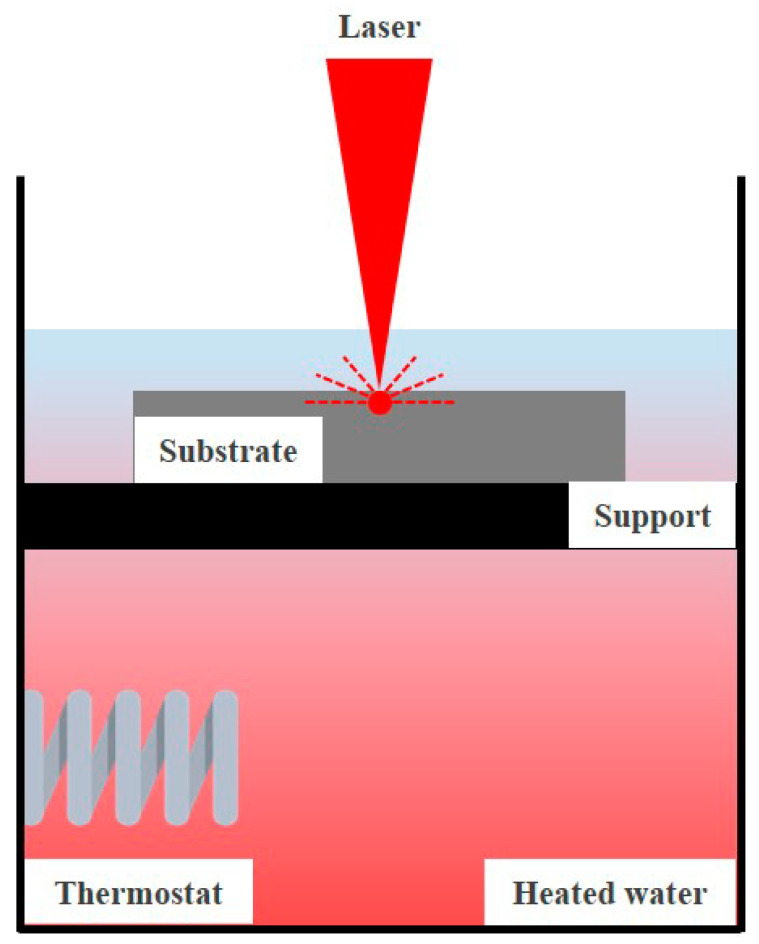
Schematic diagram of HULBM process.

In this study, we analyzed the cooling effect using water in a container at different temperatures. The cooling effect of water can reduce the thermal deformation of the parts and improve the quality of the parts. Additionally, the productivity of the parts can be improved by shortening the production time of the parts. However, the temperature of water will be increased due to the heat conduction of the material and the laser heat source if the postprocess of the parts continues for a long time. We attempted to analyze the characteristics of ULBM with water playing a cooling role in HULBM. Figure 2 illustrates a schematic of the ULBM process with the thermostat of the heating system in a water container. The water temperature increased when the process was performed for a long time. Because of the increased water temperature caused by the laser heat source, water’s efficiency as a coolant decreased. By comparing the water temperatures of 20 °C and 40 °C, it was verified that the water temperature affected the results of ULBM and heated underwater laser beam machining (HULBM). This study attempted to determine whether water can act as a coolant when the water temperature increases and analyze the effect of increasing water temperature on the length of the HAZ layer.

## 2. Materials and Methods

### 2.1. Equipment Specification

**Figure 3:**
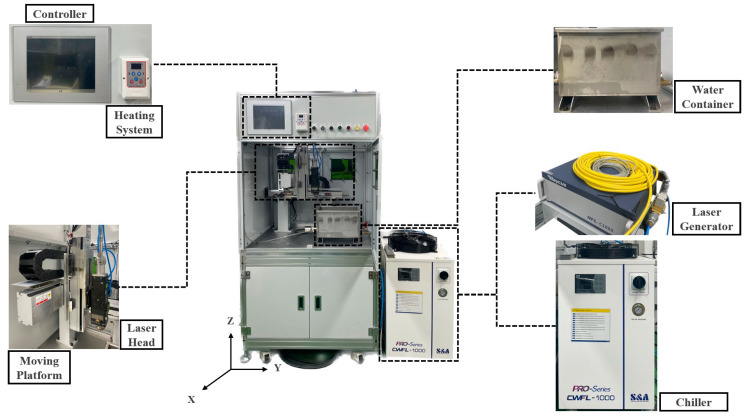
Experimental setup.

The equipment comprised a laser heat source, a water container, a focusing head, a moving platform, and a heating system. Figure 3 presents a schematic of the experimental equipment. A 1080 nm wavelength fiber laser (RFL-C1000-CW, Raycus Co., Ltd., Wuhan, China) was the laser source. The maximum output power was 1000 W with ±1.5% stability, and the output power tunability was from 10 to 100%. In addition, a fiber laser chiller (CWF-1000BN, S&A, Guangzhou, China), which can prevent unnecessary heat loss waste and maintain a constant laser wavelength, was adopted. The evaporator had a capacity of 15 L and a maximum flow rate of 70 L/min, and the temperature stability was ±0.5 °C. A water container with dimensions of 500 × 280 × 220 mm (x, y, z) was employed to minimize the rust from utilizing stainless-steel materials. The laser heat source was delivered to the surface of the 304-stainless-steel substrate by focusing the laser beam through a lens with a focal length of 300 mm, which generated a 0.5 mm diameter spot on the surface. A 304-stainless-steel substrate with dimensions of 200 × 200 × 15 mm (x, y, z) was utilized in the water container, which was fixed on the platform in the x–y plane. Compared to Inconel 718 and Ti-6Al-4V substrates, the thermal conductivity of a stainless-steel substrate is low. Additionally, the corrosion resistance of 304 stainless steel 304 is higher than that of Inconel 718 and titanium. Based on these reasons, 304-stainless-steel material was selected to analyze the cooling effect of water. The moving platform comprised a human–machine interface (HMI) controller with a programmable logic controller. A digital thermostat (DH-5562A1-CA, dhesys Co., Ltd., Busan, Republic of Korea) was adopted as the heating system. The heating system comprised a controller that could be set to a minimum temperature below 0 °C and a maximum temperature above 100 °C. The temperature of water was maintained within the 1–30 °C range.

### 2.2. Experiment Composition

To compare and analyze the HAZ produced at different temperatures, different temperatures of water were selected in the water container. The laser beam was scanned along the surface to produce a single line of 30 mm on the sample by utilizing various processing parameters. Table 1 presents the LBM, ULBM, and HULBM parameters of the experimental setup. The distance between the laser heat nozzle and the surface of the substrate was fixed at 10 mm to maximize the efficiency of the laser power reaching the substrate. Depending on the water temperature, the laser power decreases in ULBM and HULBM because of absorption and transmission. To set the laser power to reach the substrate surface in the same experimental setup, the laser power was adjusted to suit the situation. A generally known laser power efficiency calculation was adopted because the calculation differs depending on the experimental conditions. When using the heating system, the water temperature was set to 40 °C. When the water is higher than 40 °C, bubbles are generated as the water heats up and smoke produced by the heating of water can interfere with the laser path. The temperature of the water was maintained within ±2 °C. For example, if the water temperature was set to 50 °C, the heating system would turn on when the water temperature was lower than 48 °C.

In contrast, the heating system would turn off when the water temperature was higher than 52 °C. As the heating system was not used, the water temperature was set to 20 °C. This is because, when the temperature of the water is lower than 20 °C, the water can solidify into ice and the experimental setup may be changed. The experiments were repeated more than three times under the same conditions to compare the differences among the three cases.

### 2.3. Heating System

**Figure 4:**
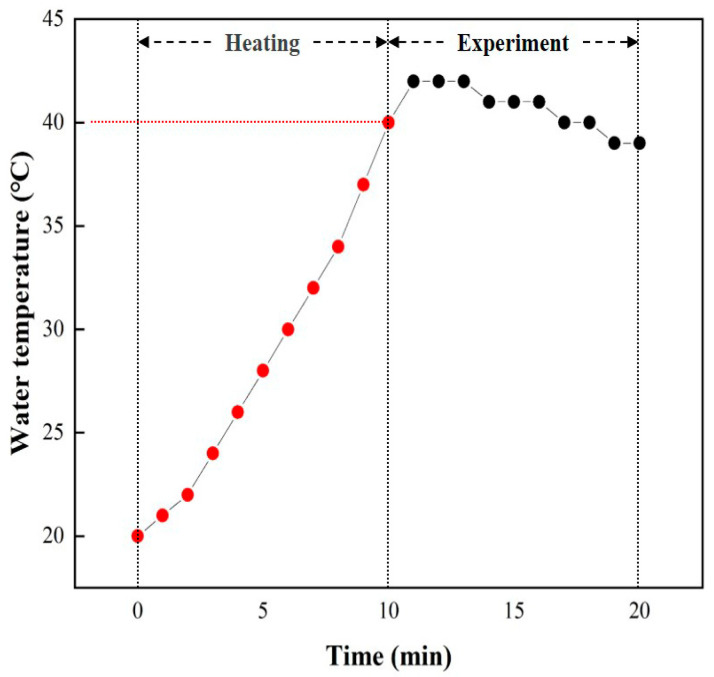
Graph of water temperature change.

The heating system required time to heat the water before the experiment was conducted. When the heating system was switched on, the water was heated and the heat was gradually transferred to the water surface by the thermostat. Finally, the heating system was turned off when the set water temperature was reached. Figure 4 presents a graph of the time required to heat the water. As mentioned in the experiment composition, the water temperature, using the heating system, was set to 40 °C in HULBM. The maximum water temperature due to deviation was 42 °C, and the minimum was 38 °C. It took about 10 min to heat the water until it reached 40 °C.

## 3. Results

### 3.1. Surface Analysis

Four water layer thicknesses were adopted in this experiment (Table 1). Before the laser was turned on, the three-axis (X, Y, Z) feed system was placed for the experiments with the HMI controller. The laser was turned on while the process was conducted in the direction and length set by the feed system. Table 2 presents the results of the experiments comparing the three conditions. A clear scanned line can be observed for the 0 mm and 1 mm water layer. However, an unprocessed scanned line was observed for the 2 mm water layer, and no distinct scanned line was observed for the 3 mm water layer. It is estimated that it would be impossible to observe the scanned line if the water layer thickness was greater than 5 mm. This is because the larger the water layer thickness, the greater the amount of laser heat source absorbed and reflected by the water. Consequently, the laser power efficiency is reduced. This suggests that it is important to set an appropriate water layer thickness.

**Figure 5:**
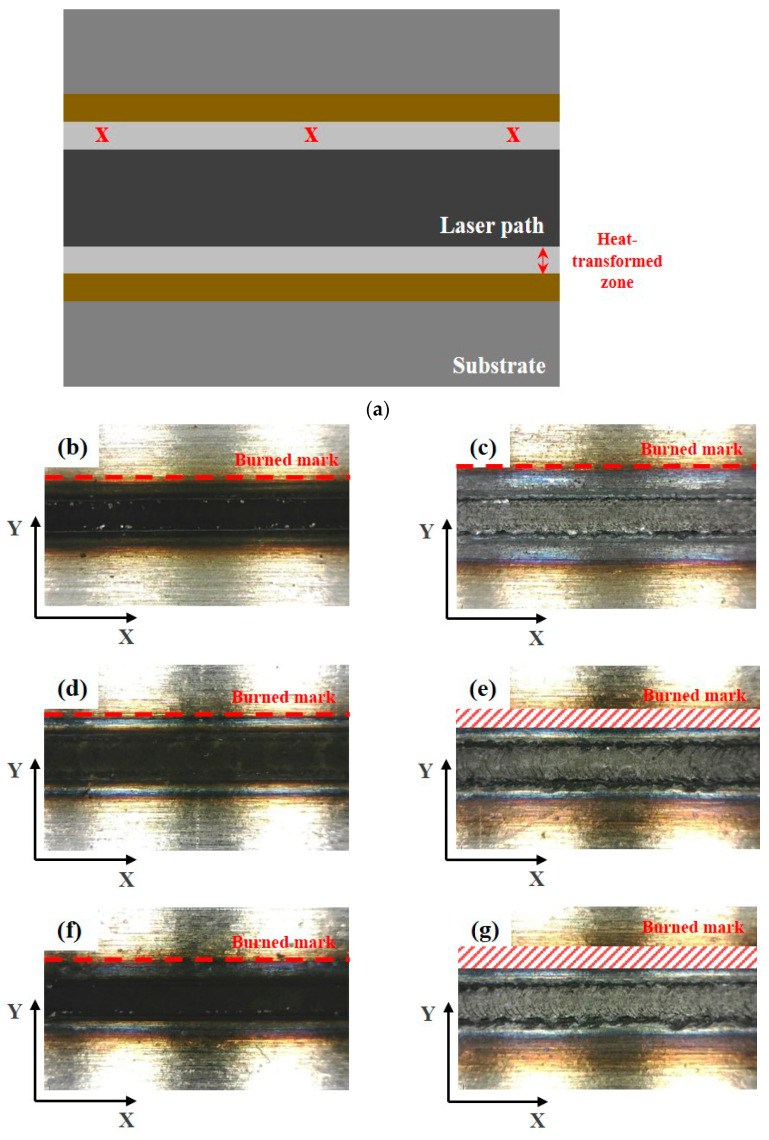
Schematic of scan line plan view and three points to measure heat-transformed zone (**a**). Microscope image of laser scan line during LBM at 200 W (**b**) and 400 W (**c**). Microscope image of laser scan line during ULBM at 200 W (**d**) and 400 W (**e**). Microscope image of laser scan line during HULBM at 200 W (**f**) and 400 W (**g**). Except for water temperature, the remaining experimental conditions were the same.

**Figure 6:**
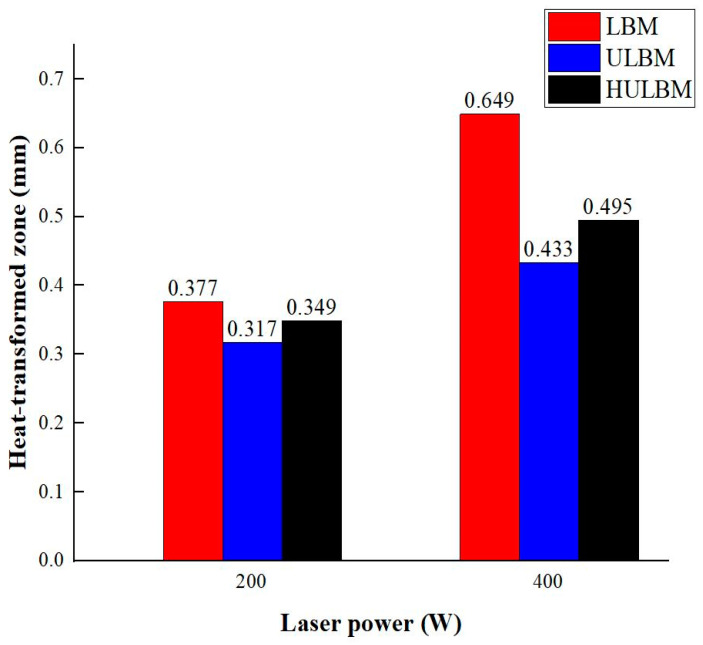
Graph of heat-transformed zone measurement differences in LBM, UBLM, and HULBM.

Figure 5 presents an analysis of the distance between the heat-transformed zones. The heat-transformed zone was measured from the end of the laser path to the edge, where it began to be charred by heat. Based on the experimental results, the average length between the heat-transformed zones was calculated by measuring at three random locations. When the laser power was 200 W, the heat-transformed zones were 0.377, 0.317, and 0.349 mm in LBM, ULBM, and HULBM, respectively. When the laser power was 400 W, the heat-transformed zones were 0.649, 0.433, and 0.495 mm in LBM, ULBM, and HULBM, respectively. Figure 6 presents the graph illustrating the results of the difference in the heat-transformed zone measurement in LBM, ULBM, and HULBM. The cooling effect of water on the heat-transformed zone was verified by comparing LBM and ULBM. In HULBM, the difference in the heat-transformed zone from ULBM was smaller than that from LBM. This means that heated water at 40 °C had a cooling effect in HULBM. In addition, when comparing the laser powers of 200 and 400 W, water affected the heat-transformed zone more. This implies that the deformation of the substrate by the laser heat source was reduced, suggesting that the mechanical properties of these parts can be improved [12,13].

### 3.2. Depth Analysis

**Figure 7:**
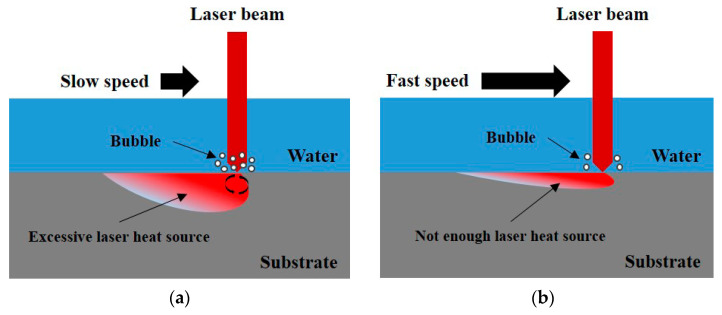
Schematic of characteristics of feed system with slow speed (**a**) and fast speed (**b**).

Figure 7 presents a schematic of the characteristics of the feed system, which were compared at different speeds. In Case A, the feed system in which the laser head was located was fast, and the laser heat source transferred to the surface of the substrate was insufficient. Therefore, ULBM was not performed because there was almost no convective phenomenon on the surface between the water and the laser beam. In Case B, the feed system was slow. An excessively large amount of laser heat was transferred to the surface of the substrate, making precise processing difficult. In addition, the number of bubbles generated by the heating water increased, which interfered with the current of the laser beam. Therefore, considering these two cases, the transfer speed of the appropriate feed system is important.

**Figure 8:**
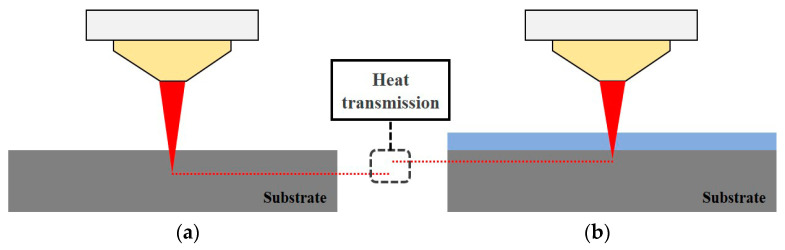
Schematic diagram of the heat transmission difference in air (**a**) and underwater (**b**).

Figure 8 presents a schematic of the heat transmission of the laser beam in air and underwater. It was possible to process a more precise scan line of the laser beam during ULBM than during LBM. This is because the inside of the substrate was affected by the cooling effect of water in ULBM, even if the laser power efficiency reaching the surface of the substrate was the same as in LBM. This means that it is possible in ULBM, such as when precise processing is required or when the shape is complicated, unlike in LBM. However, when the layer thickness of the water was greater than 3 mm, the amount of the laser heat source absorbed by the water increased. Therefore, the layer thickness of the water is important not only for the surface of the substrate, but also for the depth of the process [14,15].

### 3.3. HAZ

**Figure 9:**
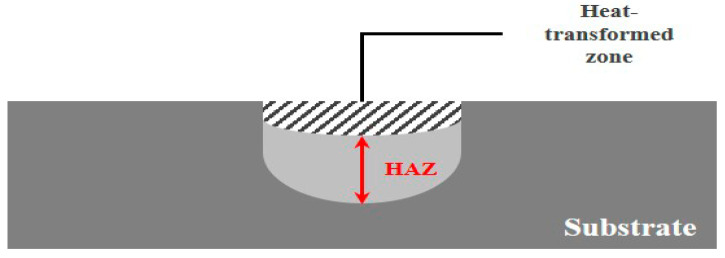
Schematic of heat-affected zone measurement.

**Figure 10:**
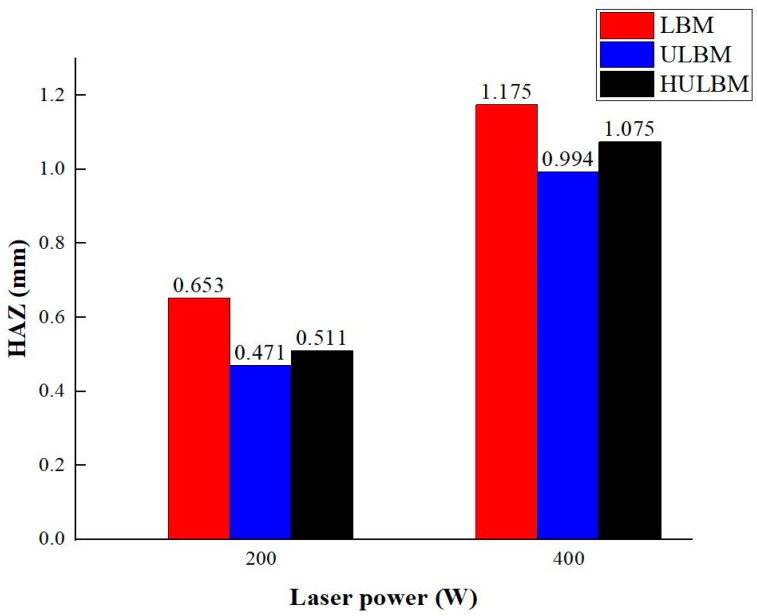
Graph of heat-affected zone measurement in LBM, ULBM, and HULBM.

Figure 9 illustrates the analysis of the length of the heat-affected zone. The HAZ was measured from the end of the heat-transformed zone to the edge, where the laser heat source deformed the structure of the base metal. When the laser power was 200 W, the HAZ was 0.653, 0.471, and 0.511 mm in LBM, ULBM, and HULBM, respectively. When the laser power was 400 W, the HAZ was measured to be 1.175, 0.994, and 1.075 mm in LBM, ULBM, and HULBM, respectively. Figure 10 presents a graph of the results of the difference in HAZ measurements in LBM, ULBM, and HULBM. Compared to LBM and ULBM, the cooling effect of water in the HAZ was verified. In HULBM, the difference between the HAZ in ULBM was smaller than that in LBM. This means that water heated at 40 °C had a cooling effect in HULBM, like the result of the heat-transformed zone difference. However, when the laser power was 200 W, the heat-transformed zone was not significantly affected. This is because different laser powers are required depending on the thickness of the base metal. This means that the thicker the 304 stainless steel, the more laser power required to reach the surface of the substrate. It is important to adjust the laser power according to the size of the base metal [16,17].

### 3.4. Vickers hardness

**Figure 11:**
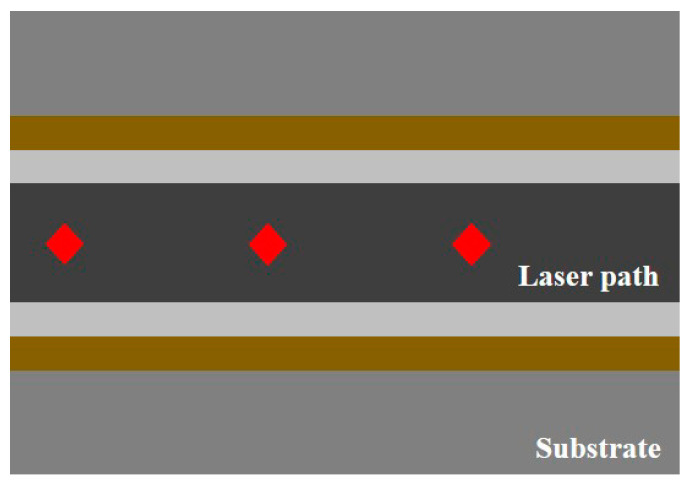
Schematic of scan line plan view and three points to measure Vickers hardness. Vickers hardness was measured at 0.5 kgf load.

Figure 11 illustrates the analysis of the Vickers hardness measurements in LBM, ULBM, and HULBM. The Vickers hardness was measured in the heat-transformed zone using a laser path. Based on the experimental results, the average Vickers hardness was calculated by performing measurements at three random locations. The Vickers hardness measurements are presented in Table 3. By comparing LBM and ULBM, the cooling effect of water on the Vickers hardness was verified. In HULBM, the Vickers hardness difference from that in ULBM was smaller than from that in LBM. Because the Vickers hardness was higher in ULBM and HULBM than in LBM, the material could be harder and stronger against external forces. In addition, the process efficiency of materials can be improved by improving their resistance.

**Figure 12:**
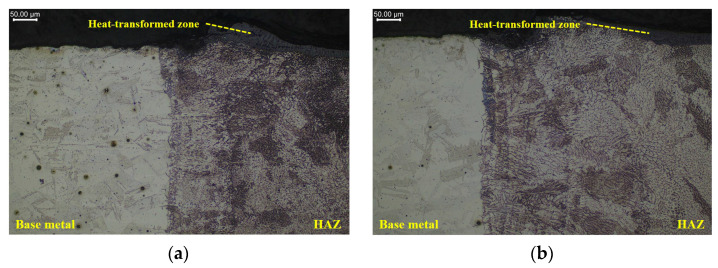
Microscope image of microstructure in LBM (**a**) and ULBM (**b**) at 400 W.

Figure 12 illustrates the microstructure of the HAZ during LBM and ULBM. Based on the results, the width of the HAZ was the largest on the top surface and gradually decreased as the distance from the top surface increased. In addition, it was confirmed that the length of the HAZ layer was shorter than that of LBM in ULBM. The distance and density between the particles in ULBM were smaller and denser than those in LBM. The measurement of Vickers hardness was affected by this result.

When the number of particles generated by the laser heat source increased, shear was easily generated owing to dislocations. Even if the laser power reaching the surface of the substrate was the same, the density of the particles increased more in ULBM and HULBM than in LBM because of the cooling effect of water. Because of this result, shear was not easily generated in ULBM and HULBM because of the densification of particles compared with LBM [18].

## 4. Conclusions

The following conclusions were derived based on the experimental improvements in the ULBM and HULBM compared with the LBM of 304 stainless steel.

When the water layer thickness was >2 mm, the laser scan line did not appear. In contrast, a laser scan line appeared when the thickness of the water layer was 1 mm. In addition, the speed of an appropriate feed system is important.

The lengths of the heat-transformed zones in ULBM and HULBM were improved by approximately 33.28% and 23.73%, respectively, compared with that in LBM at 400 W. The process did not perform properly because of the insufficient laser power required at 200 W.

The depth of the HAZ in ULBM and HULBM was improved by about 15.40% and 8.51% compared to LBM at 400 W. The mechanical properties were improved by arresting the deformation of the structure using a laser heat source.

The Vickers hardness was higher in ULBM and HULBM than in LBM. The machined stainless-steel material could be applied in various industries because its abrasion resistance, corrosion resistance, and impact resistance were improved by external forces.

## Figures and Tables

**Table 1:** TLBM, ULBM, and HULBM parameters of experimental setup.

Material	LBM	UBLM	HULBM
Laser power (W)	200, 400	200, 400	200, 400
Scan speed (mm/s)	2	2	2
Water temperature (°C)	N/A	20	40
Water depth (mm)	N/A	1	1

**Table 2:** Results of water layer thickness on surface.

Water Layer Thickness (mm)	Surface of Substrate	Characteristic of the Line
0	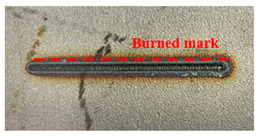	Clear scanned line
1	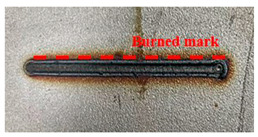	Clear scanned line
2	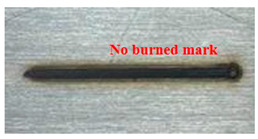	Unprocessed scanned line
3	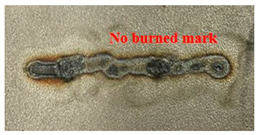	Indistinct scanned line

**Table 3:** Measurement of Vickers hardness in LBM, ULBM, and HULBM at 400 W.

Type	Hardness (HV)
LBM	212.8 ± 2.0
ULBM	240.2 ± 3.9
HULBM	226.0 ± 1.6

## Data Availability

Our resulting data can be obtained from the corresponding author upon request.
